# Acceleration of tendon–bone healing of anterior cruciate ligament graft using intermittent negative pressure in rabbits

**DOI:** 10.1186/s13018-017-0561-8

**Published:** 2017-04-18

**Authors:** Zhengming Sun, Xiaoqing Wang, Ming Ling, Wei Wang, Yanhai Chang, Guang Yang, Xianghui Dong, Shixun Wu, Xueyuan Wu, Bo Yang, Ming Chen

**Affiliations:** 1 Department of Orthopaedics of the Shaanxi Provincial People’s Hospital, Xi’an, 710068 Shaanxi China; 2 Department of Child Preventive Health Care of the Shaanxi Provincial People’s Hospital, Xi’an, 710068 Shaanxi China; 3 Department of Pathology and Laboratory Medicine, Loma Linda University Medical Center, Loma Linda, CA 92354 USA

**Keywords:** Anterior cruciate ligament, Negative pressure, Tendon–bone healing

## Abstract

**Background:**

The purpose of this study was to test effects of negative pressure on tendon–bone healing after reconstruction of anterior cruciate ligament (ACL) in rabbits.

**Methods:**

Hind legs of 24 New Zealand White rabbits were randomly selected as negative pressure group and the contralateral hind legs as control. Reconstruction of the ACL was done. Joints of the negative pressure side were placed with drainage tubes connecting the micro-negative pressure aspirator. Control side was placed with ordinary drainage tubes. Drainage tubes on both sides were removed at the same time 5 days after operation. After 6 weeks, joint fluid was drawn to detect the expression levels of interleukin-1β (IL-1β) and tumor necrosis factor-α (TNF-α); at the same time, femur–ligament–tibia complex was obtained to determine tendon graft tension and to observe the histomorphology, blood vessels of the tendon–bone interface, and expression of vascular endothelial growth factor (VEGF).

**Results:**

The maximum load breakage of tendon graft was significantly greater in the negative pressure group than in the control group (*P* < 0.05). Histological studies of the tendon–bone interface found that there was more new bone formation containing chondroid cells and aligned connective tissue in the negative pressure group than in the control group. Expression of VEGF was higher in the negative pressure group than in the control group (*P* < 0.01). Content of IL-1β and TNF-α in synovial fluid is lower in the negative pressure group than in the control group (*P* < 0.01).

**Conclusions:**

Intermittent negative pressure plays an active role in tendon–bone healing and creeping substitution of ACL reconstruction in the rabbits.

## Background

Anterior cruciate ligament (ACL) reconstruction using soft tissue autograft in bone tunnel has become popular in the past 10 years [1–3]. Tendon–bone healing of the graft at the bone tunnel is a main concern when using a tendon graft for ACL reconstruction. Because tendon graft and host bone tunnel are two types of tissue, tendon–bone incorporation is complex and slow. It is noted that the incidence rate of failures after ACL reconstruction procedures is approximately 5–25% [4, 5]. The causes of ACL reconstruction failure mainly include technical errors, biological failure, and traumatic injury. Surgical technique-related errors are the most common cause of relapsing instability after ACL reconstruction, accounting for 77 to 95% of all cases of ACL failure [6]. However, the failure of tendon–bone healing is one of the most important biological failures and should not be ignored. Therefore, firm tendon healing is one of the main factors to ensure good clinical outcome [7]. Thus, it is important to stimulate biological healing between the graft and bone tunnel for functional exercises, sports, and daily activities as early as possible.

Currently, there are many methods of promoting tendon graft and the bone tunnel healing, such as the use of platelet-rich plasma, growth factors, stem cells, scaffolds, periosteal flap, and mechanical loading [8–13]. Generally, these recent results have significantly enriched the theory of the healing of soft tissue graft with the bone tunnel in the animal model. However, most of these technologies have high cost and their clinical efficacy and safety still need to be discussed.

In recent years, the negative pressure is confirmed to have a unique advantage in promoting the wound and bone healing [14–17]. So far, there is no literature discussing the effects of negative pressure on tendon–bone healing after reconstruction of ACL. In the present study, negative pressure technology was performed in an animal model of ACL to evaluate the effects of negative pressure on tendon–bone healing of ACL.

## Methods

### Experimental design

All experimental procedures on animals were performed under the guidelines of animal ethics standard regulations approved by the biomedical ethics committee of medical college of Xi’an Jiao Tong University. Twenty-four skeletally mature male New Zealand White rabbits (age, 10–12 months; body weight, 2.9–3.5 kg) were used in this study. All experimental animals were provided by the experimental animal center of Xi’an Jiao Tong University (Production license number SCXK [Shaanxi] 2007-001; the use license SYXK [Shaanxi] 2007-003). All selected experiment animals had no joint swelling, deformity, and limp or abnormal anterior drawer test and Lachman test, and the lateral stress tests were performed on both knee joints and all were negative. Both hind legs were used in each rabbit. On each rabbit, the experimental side leg was randomly selected by coin tossing, and the other side leg was as control.

### Surgical procedure

Rabbits were anesthetized with an intravenous injection of 3.3% pentobarbital sodium (1 mL/kg) (Beijing, China). Both legs were then shaved and aseptically prepared for surgery. The rabbits with obvious joint cavity effusion and synovitis were excluded. On the experimental knee of the animal, an anterolateral skin incision was made and ACL was completely cut off; then, manual examination was used to confirm anterior subluxation of the tibia. After that, the semitendinosus tendon was identified and harvested. Both ends of the tendon were sutured with silk thread and 8-cm-long threads were retained from both graft ends as the traction line; then, the tendon was stretched for 10 min and soaked in physiological saline. According to the diameter of the semitendinosus tendon, a 2.0-mm drill tunnel was made at the intercondylar spine of the tibia and the lateral femoral condyle at the footprints of the normal ACL. Next, the autograft was pulled through the bone tunnels and the graft ends were fixed to the tunnel exits with sutures tied over the neighboring periosteum.

The anterior drawer test and Lachman test were used to check the stability of these knee joints. Then, the joint cavity was flushed, placed with the drainage tube, the wound was closed, and the drainage pipe was connected with a micro-negative pressure aspirator. The negative pressure (50 kPa, 30 min each time, two times a day) was applied after the recovery from anesthesia; the drainage tube was pulled out 5 days after the procedure. The same procedure was applied on the control lower limb but no negative pressure, and the drainage tube was pulled out at the same time with the experimental group. Each rabbit received muscular injection of 800,000 U penicillin sodium for six consecutive days after operation. Postoperatively, the rabbits were returned to their cages and bear full weight. Postprocedural knee joint infection was observed in 1 rabbit, and a total of 23 rabbits (46 knee joints) were involved in the following study. The negative pressure group had a total of 13 left and 10 right knees, on the contrary in the control group.

### Acquiring specimens

The rabbits were killed at 6 weeks by air embolism after the procedure. The previous surgical wounds were opened; the knee synovial fluid was obtained and stored at −80 °C. A knee specimen including a 60-mm-long tibia and a 50-mm-long femur was collected from each rabbit. For each specimen, the attached soft tissues were cut off from the graft, suture-tied periosteum also were cut off from outside of the drill tunnel, wrapped in gauze, moistened with physiologic saline solution, and immediately sent for biomechanical testing.

### Biomechanical testing

The tibia and the femur were separately embedded in 20 × 20 × 50 mm rectangular aluminum tubes using polymethylmethacrylate resin [18]. The prepared femur–graft–tibia specimens were attached to a microcomputer-controled electronic universal testing machine (WDW-100, Shanghai, China) for tensile test. The loading rate was 5 mm/min and recorded loading for ACL rupture or pulled out from bone tunnels.

### Histologic observation

Each graft-tibia (or femur) specimen was cut randomly at 5 mm below the tibial tubercle or 1 cm supracondylar femur for histological observation. The samples were sectioned parallel to the longitudinal axis of the tibial (or femur) tunnel. Specimens that the graft pulled out from tunnel were excluded. The specimen was then soaked in a 10% buffered formalin solution. After decalcification, the specimens were sliced through the longitudinal axis of the bone tunnel. After the specimens were being trimmed to 1 cm × 0.5 cm × 0.5 cm sizes, they were cast in a paraffin block and were cut into 5-μm-thick slices; then, they were dewaxed in xylene, rehydrated through a graded ethanol series, washed with phosphate-buffered saline, and stained with hematoxylin and eosin. The histomorphology and vascellum of the tendon–bone interface were observed, and the average number of vascellum was counted with a light microscope for 10 visions of each slice.

### Detection of interleukin-1β and tumor necrosis factor-α in synovial fluid

Concentrations of interleukin-1β (IL-1β) and tumor necrosis factor-α (TNF-α) were measured by enzyme linked immunosorbent assay (ELISA), using Quantikine ELISA kits (Shanghai, China).

### Immunohistochemical

Immunohistochemical study for vascular endothelial growth factor (VEGF) was carried out by using an avidin–biotinperoxidase complex method and a Histostain SP kit (ZymedLaboratory Inc., San Francisco, CA) according to the manufacturer’s instructions.

Before treatment with the primary antibody, endogenous peroxidase was removed by incubation in 3% H_2_O_2_, antigen retrieval was achieved by using trypsin for 5 min, and nonspecific bindings were blocked by treatment with 10% nonimmune normal goat serum at room temperature for 10 min. Human hepatoma tissue was used as a positive control. The secondary antibody of goat anti-rabbit was mixed in incubator temperature for 60 min and was stained with 3,3N-diaminobenzidine. The specimens were then counterstained with hematoxylin.

For each specimen, two blinded pathologists were responsible for counting the total vascular endothelial cells and VEGF-positive in the tendon–bone interface under 10 high-power fields (Olympus; magnification ×400). They then calculated the average number per specimen. Finally, the percentage of VEGF-positive was calculated. Immunoreactivity was graded semi-quantitatively by considering the intensity (0 = negative staining, 1 = light staining, 2 = moderate staining, 3 = strong staining) and percentage of the staining. A histological score was gained from each sample, which was obtained by applying the following formula: total score = (staining intensity × staining percentage)/10.

### Statistical analysis

Statistical analysis was carried out using SPSS17.0 software package (SPSS Co, Chicago, IL). Student’s *t* test was used to evaluate differences between numeration data. Ranked data was assessed using nonparametric Wilcoxon rank sum test. All statistical tests were two-sided, and *P* values less than 0.05 were considered statistically significant.

## Results

### Biomechanical evaluation

In the negative pressure group, the femur–graft–tibia complexes underwent rupture at the body part of the graft in 21 cases, and in 2 cases they were pulled out from the bone tunnels. In the control group, the femur–graft–tibia complexes underwent rupture at the body part of the graft in 18 cases, and in 5 cases they were pulled out from the bone tunnels. Tensile results showed that the force of complete rupture or pulled out from the bone tunnels was significantly higher in the negative pressure group than in the control group (*P* < 0.05, Table 1).

**Table 1:** The tension resulting in full rupture of the tendon graft or pull out

Group	Sample	Maximum tension (N)	*P*
Negative pressure	23	21.71 ± 2.59	0.047
Control	23	20.27 ± 2.39	

### Histological observation

**Fig. 1:**
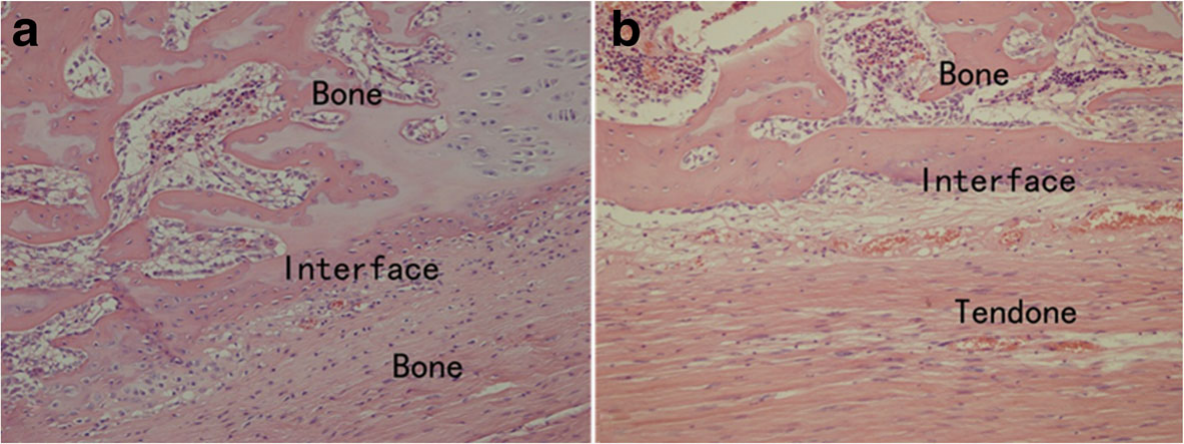
Histology of the tendon–bone interface, H-E stained ×200. **a** There was aligned connective tissue, newly formed woven bone, and cartilage in the tendon–bone interface of negative pressure group. **b** There was less fibrous connective tissue and aligned chondroid cells in the tendon–bone interface of control group

**Fig. 2:**
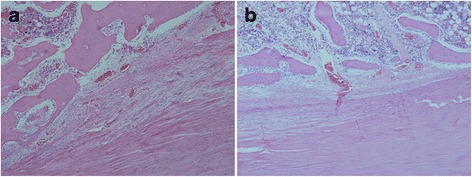
Vascular distribution of the tendon–bone interface, H-E stained ×200. **a** Blood vessels rich in the tendon–bone interface of negative pressure group. **b** Blood vessels sparse in the tendon–bone interface of control group

There were more new bone formation containing chondroid cells and aligned connective tissue between the interface of the bone tunnel and the tendon in the negative pressure group at week 6 (Fig. 1a). In the control groups, there was less fibrous tissue, and very little new bone formation containing chondroid cells in the tendon–bone interface (Fig. 1b). The tendon–bone interface had a lot of vascular distribution in the negative pressure group (Fig. 2a), but vascular structure on the tendon–bone interface was sparse in the control group (Fig. 2b). The number of blood vessels was significantly greater in the negative pressure group than in the control group, and the difference was statistically significant (*P* < 0.05, Table 2).

**Table 2:** The number of blood vessels in the tendon–bone interface

Group	*n* (sum of femur and tibia)	The number of blood vessels	*P*
Negative pressure	42	15.21 ± 1.36	0.000
Control	36	9.29 ± 1.97	

### Detection of IL-1β and TNF-α in synovial fluid

Concentrations of IL-1β and TNF-α are listed in Table 2. Compared with the control group, content of IL-1β and TNF-α in synovial fluid decreased significantly in the negative pressure group, and the difference was statistically significant (*P* < 0.05, Table 3).

**Table 3:** The level of expression of IL-1β and TNF-α in synovial fluid

Group	Sample	The content of IL-1β (ng/L)	The content of TNF-α (ng/L)
Negative pressure	23	10.94 ± 1.64	12.21 ± 1.36
Control	23	12.96 ± 1.86	18.29 ± 1.97
*P*		0.002	0.000

### Immunohistochemistry results

**Fig. 3:**
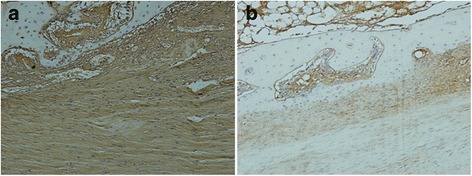
Expression of VEGF in the tendon–bone interface, Streptavidin/peroxidase method, DAB color, ×200. **a** Expression of VEGF in the tendon–bone interface of negative pressure group. **b** Expression of VEGF in the tendon–bone interface of control group

The vascular endothelial cells in the tendon–bone interface of the negative pressure group (Fig. 3a) show strong and diffuse staining, whereas the staining in the control group is only weak and focal (Fig. 3b). The expression intensity of vascular endothelial growth factor was significantly higher in the negative pressure group than in the control group, and the difference was statistically significant (*P* < 0.05, Table 4).

**Table 4:** The expression level of VEGF in the tendon–bone interface

Group	*n* (sum of femur and tibia)	Total score	*P*
Negative pressure	42	11.45 ± 1.35	0.000
Control	36	5.67 ± 1.06	

## Discussion

ACL reconstruction using autograft for reconstruction materials has been the mainstream choice of ACL rupture. Early connection of the tendon–bone interface is not strong. Therefore, the tendon graft and bone tunnel firm healing are one of the major factors affecting the success of ACL reconstruction with autologous tendon or allogeneic tendon [19]. In the present study, low intensity, intermittent, negative pressure was maintained in the knee joint of rabbits under ACL reconstruction. We studied the effects of negative pressure on the tendon–bone interface healing by studying the histological changes of the tendon–bone interface, graft strength, expression of VEGF, and content of IL-1β and TNF-α in synovial fluid.

We refer to previous studies in the selection of negative pressure and maintenance time in this study. Yang demonstrated that bone marrow-derived stroma cells showed a typical appearance of osteoblast after 2 weeks of induction by intermittent negative pressure (50 kPa, 30 min/time, and twice daily) [14]. In Zhang’s study, negative pressure was intermittently administered for 4 weeks (pressure 50 kPa, 30 min/time, and twice daily) and this negative pressure was able to promote the regeneration of bone in the study of the repair of rabbit skull defects [16]. According to these results, although good results can be obtained by maintaining negative pressure for a long time, we believe that the longer the drainage tube placement is, the higher the risk of infection will be. Infection is a devastating consequence for the joint, and there was one case of joint infection in our study. Therefore, we maintained joint intermittent negative pressure (pressure 50 kPa, 30 min/time, and twice daily) for 5 days to reduce risk of infection in our study.

Rally measurement results show that maximum load breakage of tendon graft was significantly greater in the negative pressure group than in the control group. Histological studies of the tendon–bone interface found that there were more chondroid cells containing new bone formation and aligned connective tissue in the negative pressure group than in the control group. Immunohistochemistry showed that expressions of VEGF of osteoblasts were higher in the negative pressure group than in the control group. These results confirmed that intermittent negative pressure may promote tendon–bone healing. Several possible mechanisms explain these observations.

First, this may be related to mechanical stimulation, hypoxia under negative pressure. Mechanical stimulation, one of the basic stimuli in the process of cell growth, plays an important role in cell differentiation and proliferation; many experiments in vitro have confirmed that mechanical stimulation may induce osteoblastic differentiation [20–22]. Negative pressure environment in the joint is bound to cause tissue hypoxia, and one study found that hypoxia could increase the bone forming ability of bone marrow mesenchymal stem cell in rat [23]. Some studies have confirmed that low intensity intermittent negative pressure can successfully induce the differentiation of human bone marrow mesenchymal stem cells into osteoblasts [14], inhibit bone absorption, and promote ossification [24].

Secondly, promoting tendon–bone healing by negative pressure may be related to vascularized and high expression of VEGF of osteoblasts in the tendon–bone interface. Endothelial cell migration is limited in the absence of negative pressure, while the negative pressure may promote the proliferation and migration of vascular endothelial cells, and facilitates the formation of vessels [25]. VEGF can increase the microvascular permeability, can provide nutrition for the newly formed capillary network and cell growth, and can change extracellular matrix, thereby promoting microvascular ingrowth within the organization. Expression of VEGF mRNA of human bone marrow mesenchymal stem cells was significantly high in vitro under negative pressure [24], which is similar to the findings in this study. We speculate that more bone marrow mesenchymal stem cells intermittently move into the tendon–bone interface of intra articular and transform to osteoblasts when intermittent negative pressure is maintained in the joint cavity. At the same time, the negative pressure may suppress bone absorption on the tendon–bone interface, promote bone formation, and as such promote the tendon–bone interface healing. In addition, the content of VEGF in the tendon–bone interface may increase under intermittent negative pressure; with the increasing of the number of vascular endothelial cells, the more vascularization is prone to form in the tendon–bone interface, thus conducive to the healing of the tendon–bone interface.

Thirdly, we found that the content IL-1β and TNF-α in synovial fluid were lower in the negative pressure group than in the control group. Inflammatory cells and inflammatory factors abnormally increase in synovial fluid after cruciate ligament reconstruction and are not conducive to the healing of the ligament. Synovial fluid can enter the gaps between the tendon and bone tunnel after operation. The inflammatory cytokines in synovial fluid, such as IL-1, TNF-α, and matrix metalloproteinase, degrade bone tissue and inhibit the tendon–bone healing [26]. Cameron et al. also considered that the joint fluid was one of the most important factors causing osteolysis of bone tunnel and bone tunnel enlargement [27].

Wen et al. found grafted tendon healing in the tibial tunnel was inferior to that in the femoral tunnel at the tendon–bone interface after ACL reconstruction in rabbits [28]. These researchers suggested that synovial fluid is not conducive to the healing of the tendon–bone interface. Our study supports this conclusion. IL-1 may stimulate TNF-α, IL-6, IL-8, and matrix metalloproteinase secretion and aggravation of articular structure damage [29, 30]. We think that the intermittent suction can not only reduce the amount of joint fluid, especially significantly decrease level of IL-1 and TNF-α, indirectly reduce the release of the other inflammatory mediators, inhibit bone and tendon graft destruction and degradation, but also promote tendon–bone interface healing and tendon graft creeping substitution.

To our best knowledge, so far, there is no report about intermittent negative pressure technology being applied in animals under ACL reconstruction. We found that intermittent negative pressure plays a positive role in tendon–bone interface healing and tendon graft creeping substitution after anterior cruciate ligament reconstruction on rabbits. The possible explanation of these observations include the following: (1) intermittent negative pressure promotes the vascularization of the tendon–bone interface by increasing of VEGF expression; (2) the negative pressure suction reduces the contents of IL-1 and TNF-α in joint fluid and as such, inhibit bone and tendon destruction and degradation. However, more research needs to be done in the future in order to elucidate more underlying mechanisms.

## Conclusions

Although the underlying mechanisms need to be further demonstrated yet, the present study shows that intermittent negative pressure may enhance ability of the tendon–bone healing after reconstruction of ACL in rabbits.

## Abbreviations

ACL: Anterior cruciate ligament
ELISA: Enzyme linked immunosorbent assay
IL-1β: Interleukin-1β
TNF-α: Tumor necrosis factor-α
VEGF: Vascular endothelial growth factor
